# An Exploration of the Needling Depth in Acupuncture: The Safe Needling Depth and the Needling Depth of Clinical Efficacy

**DOI:** 10.1155/2013/740508

**Published:** 2013-07-09

**Authors:** Jaung-Geng Lin, Pei-Chi Chou, Heng-Yi Chu

**Affiliations:** ^1^School of Chinese Medicine, College of Chinese Medicine, China Medical University, No.91 Hsueh-Shih Road, Taichung 40402, Taiwan; ^2^Department of Rehabilitation, Tri-Servcie General Hospital, National Defense Medical Center, No. 325, Sec. 2,Chenggong Rd., Neihu District, Taipei City 114, Taiwan

## Abstract

*Objective*. To explore the existing scientific information regarding safe needling depth of acupuncture points and the needling depth of clinical efficacy. *Methods*. We searched the PubMed, EMBASE, Cochrane, Allied and Complementary Medicine (AMED), The National Center for Complementary and Alternative Medicine (NCCAM), and China National Knowledge Infrastructure (CNKI) databases to identify relevant monographs and related references from 1991 to 2013. Chinese journals and theses/dissertations were hand searched. *Results*. 47 studies were recruited and divided into 6 groups by measuring tools, that is, MRI, in vivo evaluation, CT, ultrasound, dissected specimen of cadavers, and another group with clinical efficacy. Each research was analyzed for study design, definition of safe depth, and factors that would affect the measured depths. Depths of clinical efficacy were discussed from the perspective of de-qi and other clinical observations. *Conclusions*. Great inconsistency in depth of each point measured from different subject groups and tools exists. The definition of safe depth should be established through standardization. There is also lack of researches to compare the clinical efficacy. A well-designed clinical trial selecting proper measuring tools to decide the actual and advisable needling depth for each point, to avoid adverse effects or complications and promote optimal clinical efficacy, is a top priority.

## 1. Introduction

Acupuncture is an important part of traditional Chinese medicine and has been used for millennia of years to treat various clinical disorders based on ancient Chinese medicine theory. In recent one hundred years, acupuncture has become one of the most popular complementary and alternative therapies in the world. More than 100 million citizens in the European Union make use of complementary and alternative medicine (CAM) today. According to EICCAM files, the most commonly used CAM therapies in Europe are homeopathy, acupuncture, phytotherapy (i.e., herbal medicine), anthroposophic medicine, naturopathy, traditional Chinese herbal medicine, osteopathy, and chiropractic. In 2007, almost 4 out of 10 adults had used CAM therapy in the past 12 months. Results from the 2007 NHIS found that approximately one in nine children (11.8%) used CAM therapy in the past 12 months. Between 2002 and 2007, increased use was seen among adults for acupuncture, deep breathing exercises, massage therapy, meditation, naturopathy, and yoga in the United States [1]. Given the fact of the rising incidence of chronic disease and stress-related illness in the West, along with an expanding awareness of the unwanted side effects of pharmaceutical treatment, there has been an increased utilization of acupuncture as a contemporary health care option [2]. Acupuncture is also practiced by about 40,000 physicians in Germany [3]. One of the three most commonly used methods of CAM is acupuncture in the United Kingdom [4]. There are 12%~19% of individuals who had received acupuncture treatment in Europe [5]. A practitioner in UK reported that an estimated 10.0% of the UK population had received any CAM therapy (an estimated 6.5% had used one of the five main therapies: acupuncture, homeopathy, chiropractic, osteopathy, or herbal medicine) [6]. Acupuncture points are known as specific locations of the body that are needled during acupuncture treatment. Acupuncture points are located along meridians that have been defined by ancient writings of Chinese medicine since thousands of years ago. Traditionally, acupuncture points are localized using cun (or Tong Shen Cun) as proportional measurement. Ancient writings of acupuncture guidelines also refer to anatomical landmarks to help localize the needling position. Cuns usually used in the documents are as follows [7].

### 1.1. Proportional Bone (Skeletal) Cun (B-Cun)

This method divides the height of the human body into 75 equal units. Using joints on the surface of the body as the primary landmarks, the length and width of every body part is measured by such proportions. The specific method is as follows: divide the height of the human body into 75 equal units and then estimate the length and width of a certain part of the body according to such units. One unit is equal to one cun. 

### 1.2. Finger Cun (F-Cun)

This method is based on the finger cun of the person to be measured for acupuncture point locations. 

### 1.3. Fingerbreadth (F-Breadth)

This method utilizes the width of the distal phalanx of the middle finger. This should be distinguished from the middle finger cun. 

 For example, the individual distance between nipples measures 8 cun, and the individual interscapular distance measures 6 cun. Several research reports have discussed the anatomy and physiology of acupuncture points in order to understand the therapeutic mechanism of acupuncture [39–42]. However, the actual mechanism by which acupuncture works remains controversial. The majority of these studies have been of an experimental nature or in vitro cadaver studies and lack discussions regarding needling depth. Acupuncture is generally considered to be a safe treatment. Most reported adverse events were minor complications such as needling pain, hematoma, nausea, vomiting, and fainting. Ancient Chinese literature and historical texts have also documented the adverse effects of acupuncture. There are two descriptions about the possible critical complications of acupuncture in chapter 60 of *Huangdi Neijing: Spiritual Pivot*. “Unskillful doctors may kill the patients instead of saving their lives.” “Violation of the rules in performing needling therapy will kill the patients instead of saving their lives.” Deep insertion at the acupuncture point Qupen (ST12) may cause dyspnea, cough, and even collapse of the lung. Complications in acupuncture practice may result from violations of sterile procedure and/or negligence of the practitioners. Serious side effects include cardiac tamponade [43, 44], pneumothorax [45, 46], endocarditis [47–49], hepatitis [50–52], chylothorax [53], and spinal cord injury [50, 54], and minor side effects include fainting [55, 56] and skin reactions [57–60]. Pneumothorax is the most common mechanical organ injury associated with acupuncture treatment [50, 61–64], and the related reports are from the United States [65–67], Canada [68], The Netherlands [69, 70], France [71], Norway [63], Portugal [72, 73], Denmark [74], Taiwan [75], Japan [50, 76], China [45, 77], and Hong Kong [78]. Based on the facts mentioned above, we understand that the safe needling depth for acupuncture therapy is a very important issue for clinical practice. However, there is only meager and confusing information about the safe needling depth in ancient classics of Chinese medicine and modern acupuncture textbooks. As some adverse events are preventable through preventive measures, there stands the need for urgent standardization regarding safe depth of acupuncture. We are also interested to learn if needling depth is correlated with the clinically observed therapeutic effects. In this paper, we provide a critical review of the current researches classified by the measuring tool on safe needling depth of acupuncture points as well as the therapeutic depth with clinical efficacy. 

## 2. Methods

A comprehensive search of the literature that was published from 1991 to 2013 was undertaken using the following key words: acupuncture, acupoint, needling depth, safe depth, dangerous depth, de-qi, therapeutic effect/efficacy, and their synonyms. These terms were used to search the following databases: PubMed, EMBASE, Cochrane, Allied and Complementary Medicine (AME), The National Center for Complementary and Alternative Medicine (NCCAM), and China National Knowledge Infrastructure (CNKI) databases. Additional articles were also identified from the reference list of identified articles. Chinese journals, theses, and dissertations that we thought might be relevant to our study were hand searched. We excluded the animal studies.

## 3. Results

47 studies from 1991 to 2013 were recruited into the review. As there are not many researches specific for depth of acupuncture points, we tried to include as many articles as possible. Most of the studies were retrospective, nonrandomized clinical trials without control group. The characteristics of subjects, sample size, investigated acupuncture points with the associated body region/meridians, parameters used for comparison, related factors, results and suggestions/conclusions of the researches and related factors and the results and suggestions and conclusions of the researches were summarized in the following tables classified by their measuring tool, that is, magnetic resonance imaging (MRI), in vivo subjects evaluation, computed tomography scan (CT), ultrasound, and dissected specimens of cadavers, in Tables 1, 2, 3, 4, and 5.  Table 6 depicts the investigations regarding clinical efficacy.

### 3.1. The Safe Needling Depth

#### 3.1.1. Researches Using MRI Images for Measurement of the Depths of Acupuncture Points

Magnetic resonance imaging (MRI) is a medical imaging technique used in radiology to visualize internal structures of the body in detail. MRI provides better contrast between the different soft tissues of the body compared with other medical imaging techniques such as CT scans or X-rays which makes it the more appropriate measuring tool detecting acupuncture points in specific body regions. Unlike CT scans or traditional X-rays, MRI does not use ionizing radiation.  Table 1 summarizes 14 studies (composed of 16 papers) that met the search criteria. These studies investigated 17 various acupuncture points in the head, face, chest, abdomen, and back region. Factors that may affect the measured depth including gender, body length (BL), body weight (BW), right or left side points, needling angle, body mass index (BMI), neck girth, and cun (thumb or body cun) were used for comparison. The results do not reach unanimity and contradict each other. For example, male subjects had greater safe depth only in BL18, BL19, and GV16 [8, 10, 16, 19, 21, 23]. The perpendicular depths of right side points correlated with BW, thumb cun while the depths of left side points correlated with BL, BW, BMI, and thumb cun in BL18 [8]. Needling depth correlated positively with BW, BMI, and neck girth in ST18, GB24, LR14, GB20, GV16, and GV15 [15, 16, 20, 21, 23]. On the other hand, the safe depth did not correlate with BL, BW and BMI in BL20, BL17, SP21, CV14, ST19, CV13, and CV15 [9, 11–14, 17, 18]. 

#### 3.1.2. Researches Using In Vivo Evaluation Methods in Real Subjects

We recruited 8 studies that met the search criteria and are summarized in Table 2. Under this category, two studies were specifically conducted to treat patients with low back pain [24] and intervertebral herniation of cervical spines [28]. We observed a significant correlation between the interscapular distance and the thickness of the soft tissue layer with the BMI at BL25, BL26. As a result, using proportional methods is relevant for the success of acupuncture therapy [24]. Association between nerve contact and de-qi was also discussed. The rate of median nerve penetrations by the acupuncture needle at P6 was surprisingly high, but these seemed to carry no risk of neurologic sequelae. De-qi at P6 did not depend on median nerve contact, nor did it prevent median nerve penetration which confirms the idea that acupuncture is a safe treatment method [25]. The definition of safe depth should be less than 70% of dangerous depth as suggested in most of the similar investigations [26, 27]. From the needling angle perspective, safe needling angle should be 10 degrees more than dangerous angle at 7 points from bladder meridian (1st side line) [27, 29]. The measured depth of GV14, all back bladder meridian points, and some chest points were greater than documents from ancient writings [28, 29]. The depths of all back bladder meridian points and some chest points highly correlated with body thickness and Tong Shen Cun [29]. Points of female chest had greater depth than male. De-qi depth is related to therapeutic effect; however, no correlations between the de-qi depth and electric resistance of each point in the chest and back regions were revealed [29, 31].

#### 3.1.3. Researches Using CT Scan Images for Measurement of the Depths of Acupuncture Points

One of the mainstreams of measuring acupuncture points is using images from CT scans (13 studies were recruited in this review). One study defined the T/S ratio (therapeutic depth over safety depth). The therapeutic depth was defined as the depth at which the needle is in the muscular layer of specific acupuncture point. Chen et al. suggested that the T/S ratios were between 0.67 (SP-15) and 0.88 (CV-6, CV-10). The therapeutic depth of abdominal acupoints was closer to the safe depth in overweight and older children aged 7 to 15 [32]. As for the definition of safe needling depth, it should be less than 75% of the dangerous depth [33, 35, 36], but there were two studies reported that it should be report that to be 70% of the dangerous depth [26, 27]. Depths from in vivo CT images revealed that they were greater than the ones retrieved from cadavers [26, 27]. According to the research of Lin, there were significant differences in chest points within the same sex, however, female chest points had greater depths [37]. In children subjects, the safe depths of studied points (CV-2 to CV-7 and CV-9 to CV-14) were 1.3–2.1 times deeper in the 12–15-year-old group than in the 7–9-year-old group and 1.7–3 times deeper in overweight children than in underweight children. The depths increased significantly with age and body size yet with large variations [34]. 

#### 3.1.4. Researches Using Ultrasound Images for Measurement of the Depths of Acupuncture Points

We included 2 studies under this category. Lian suggested that needling depths of acupoints BL11 to BL21 ranged from 12–40 mm and not affected by age, body sizes, and disease types. Gender and side differences also existed, and depths measured were shorter compared to ancient writings [79]. Streitberger et al. found that there was no association between the number of nerve contacts and de-qi when needling at PC6, and the mean distance from the needle tip to the nerve was 1.8 mm (standard deviation 2.2; range 0–11.3). The ultrasound has the advantage of acquiring real-time images; as a result, the authors were able to observe actual nerve contacts by the needle tip, and thus the possible complication of nerve penetration was recorded for analyses (leaving no neurological sequelae) [25].

#### 3.1.5. Researches Using Dissected Specimens of Cadavers for Measurement of the Depths of Acupuncture Points

We included 21 investigations under this category. This is another measuring method used extensively in early investigations which continues to be valuable for certain points. A few studies defined the safe depth and dangerous depth for needling, respectively. For example, five studies suggested that the safe depth should be less than 70% of dangerous depth [26, 27, 86, 89, 95, 96]. Yan et al. suggested that the safe depth of GV15, GV16, GB20, and BL1 should be 80% of the measured depth [97]. Li et al. defined the safe depths to be within 75% of the measured depths because they used in vivo CT images, which should be greater than the ones from cadavers [27]. In short, most of the authors chose 75% or 80% from their clinical experience rather than conclusive anatomical evidence. Consequently, there is no universal definition of dangerous depth, safe depth, or therapeutic depth. Wang proposed that the needling depth of ST7 and SI18 revealed no side difference, and the mean inserting depths from skin surface to sphenopalatine ganglion are 49.9 and 46.6 mm, respectively, which may change by different puncturing directions [81]. As for gender and side perspective, there was no gender difference in dangerous depth in SI14, ST12, BL1, L12, BL13, GV14, GV15, GV16, and GB20 and acupoints in back and lumbar regions [82, 84, 85, 95]. There was no side difference in dangerous depth in ST7, ST12, SI18, GV15, GV16, and GB20, 7 points from bladder meridian (2nd side line), 7 points from bladder meridian (1st side line), 28 acupoints in back and lumbar regions, and 23 chest points [26, 27, 81, 84, 95–97]. Details of each research are summarized in Table 5.

### 3.2. The Needling Depth of Clinical Efficacy

This section is composed of 11 researches. Lin had first investigated the needling depths of acupuncture points regarding de-qi in a series of researches since 1991 [29–31]. He proposed that de-qi depth was related to therapeutic effect. Depth of de-qi was greater in males and people with greater body weight except for chest points in females. He also found no correlations between the de-qi depth and electric resistance of the acupuncture points. Chen et al. also used the therapeutic depth over safety depth ratio (T/S ratio) as the indicator of therapeutic depth. There was no significant difference in the T/S ratio between genders, and the T/S ratio of these 12 acupuncture points ranged from 0.67 to 0.88 and increased significantly with body weight, age, and waist girth [32]. Groenemeyer et al. suggested that an association between de-qi and needle location exists [24]. However, Streitberger et al. found no association between the number of nerve contacts and de-qi [25]. The depth of needle penetration counted for the clinical efficacy of relief of muscle pain [102]. Deep puncturing at ST7 was more effective than routine puncturing, and the total effective rate in deep puncturing group is superior than that in shallow puncturing group [98]. Itoh et al. suggested that immediate pain relief in muscle group (deep insertion for 10 mm) was better than that in skin group (insertion for 3 mm) [102]. Deeper insertion also induced more dull sensations as compared to shallow insertions which induced more sharp sensations. In addition, needle rotation significantly increased the dull sensations [99]. Lu and Tang confirmed the various needling depths ranged from 2–12 mm as documented in Lingshu (Miraculous Pivot) used for treating irritable bowel syndrome of diarrhea [101]. 

## 4. Discussion

To our knowledge, this is the most comprehensive research to review all the studies regarding safe needling depth and clinical efficacy of acupuncture points. We tried to include researches as many as possible to provide a solid foundation for evidence based medicine in terms of advisable needling depth when performing acupuncture treatment. Evidence based medicine allows researchers or clinicians nowadays to ask a more extensive spectrum of miscellaneous research questions such as “Is acupuncture more effective than placebo?” or “Is CAM therapy along with wonted care more effective than wonted care alone?” These issues could be dealt with through appropriate study designs. 

Studies of acupuncture safety indeed need special attention to needling depth issues. For example, the relationship between the effect of acupuncture analgesia and needling direction, angle, and depth has been optimized to enhance the analgesic effect as suggested by Fan et al. The result shows that all the 3 factors are the key influences. However, studies addressed the more specific correlation between needling depth, and issues aroused from safety or clinical efficacy are still far from enough [103].

Most of the researches included in this review are retrospective in nature along with small sample size and lack randomization and control group, and most of the researches did not apply the WHO Standard Acupuncture Point Locations, let alone that they employed different measuring tools. Therefore, we observed the great inconsistency of measured needling depth among different subject groups. The inconsistency may also result from the following variables not strictly controlled in most of the researches:

1. the difference in ethnicity;
2. the age of the subjects;
3. the gender difference;
4. the subjects were not all divided in to groups by a more specific index for body sizes such as BMI;
5. most of the subjects were not grouped by their underlying condition, that is, healthy subjects or with specific medical conditions;
6. the definitions of safe needling depth, dangerous needling depth were obscure including the needling angle of specific acupuncture point and which side of the body the points were located.

All these facts lead to discrepancy in the safe depth measurements. For example, the suggested safe depths for GV16 are 27.05–45.55 mm (using MRI images) [21], 27.73–33.39 mm (using CT images) [33], 40.08 mm (using dissected specimens) [97], and 43.46–57.42 mm (using dissected specimens) [94].

In order to lift the level of acupuncture safety, it is necessary to utilize modern imaging technology to explore the safe depth of each acupuncture point all over the human body. We should take the regional and anatomical properties of different groups of points into consideration when deciding the measuring tools. For example, MRI imaging is very suitable for the acupuncture points in the abdominal and back regions due to the excellent ability to obtain soft tissue details while CT scan images are extremely helpful when detecting chest points. Ultrasound is a convenient tool which can obtain real-time images when trying to observe the clinical efficacy simultaneously, especially applied in vivo subjects. Using direct specimen dissections of cadavers is another measuring method. After frozen, anticorrosive, positioning, and dyeing process, the original elasticity and properties of the corpse tissues have been lost; the concerns which may arise from this kind of study are that specimens are drier and smaller than living human tissues. This may lead to possible inconsistency in measured needling depth. For example, Li et al. [27] and Dong et al. [26] compared the depths of 7 points from bladder meridian and found that depths measured via in vivo CT images were greater than ones from dissections of cadavers. 

The measurement of needling depth involves the detection of soft tissue mostly. Different measurement methods often result in possible discrepancies due to the characteristic of each measuring tool. For example, Fiirgaard et al. suggested that MRI is superior in estimating the volume of acoustic neuroma than CT scan and with less inter-examiner difference [104]. MRI was reported to be better in identifying suspected disease of the brain and cervical spinal cord [105]. The sensitivity for detecting bony osteolytic lesions was 51.7% for radiography, 74.7% for computed tomography, and 95.4% for magnetic resonance imaging as reported by Walde et al. [106]. Yet MRI scanogram is slightly less accurate compared with radiographic scanogram in detecting limb length differences [107].

In addition, we also found several studies discussing the divergence among different measuring tools when investigating various body tissues including adipose volume (visceral or subcutaneous), muscle thickness, bone loss, and cartilage thickness. All these body tissues are relevant to the measurement of acupuncture points. However, the results indicated that no single measuring method would be more suitable than the other one (MRI, CT, and ultrasound). There was no significant difference between CT and MRI in the measurement of adipose tissue, glenoid bone loss, hip cartilage thickness, pheochromocytoma and in vivo skeletal muscle. Ultrasound was as good as MRI in the evaluation of supraspinatus and deltoid muscle with high correlation coefficients (0.96 and 0.97, resp.). MRI was also proved to be as precise as direct cadaver measurement to evaluate adipose tissue. 

There was no difference using either ultrasound or CT for the measurement of visceral fat volume. While ultrasound was as good as CT and MRI to assess intra-abdominal adipose tissue [108–117].

Some authors compared the results of MRI/CT imaging with cadavers in measuring adipose tissue [110], skeletal muscle [115], and hyaline cartilage thickness of hips [117] and found that both MRI and CT can serve as a good reference tool in the measurement. But Stevens-Simon et al. reported that the results of their study do not support the validity of ultrasound measurement of visceral adiposity as a measure of central adiposity in postpartum teenagers [118].

Consequently, the results in terms of measuring specific body tissues using different measuring methods are ambiguous and sometimes conflicting with each other. This fact may partially explain the inconsistency of needling depth of each acupuncture point as reviewed in our study.

Some authors discussed the interobserver reliability as well. Botser et al. thought that CT was found to have higher interobserver reliability than MRI when deciding the degree of femoral anteversion [119]. MRI was again found to have less interexaminer difference in calculating the soft tissue volume [104]. Both studies pointed out that some of the measuring errors may come from the man-made technical faults.

As a result, it is very difficult to obtain a general conclusion regarding safe needling depth using these results; future researches are warranted which will be discussed later in the paper.

Some theses about using MRI for safe needling depth detection we included in this study seem to have the same sample population (number, sex ratio, age, and BMI are consistent with each other) which may result in bias in the interpretation of study results. In addition, the sample size is limited to 20 subjects which lack the power for further analysis of gender, age, and body size differences.

From the review, we learn that many factors may influence the measurement of needling depth. They include gender, age, body size (such as body length, body weight, waist girth, and BMI), which side of the body, angle of needling, and so forth. Chou et al. have reviewed part of the researches previously [120]. Among these factors, body size is always considered the most significant one which complies with our general understanding that subjects with greater body size would have greater measured depth in most of the acupuncture points. However, anatomical and structural difference exists in different human body regions when we take account of the factor like BMI. 

The measured depth in body region with frequent accumulation of fat would be highly correlated with BMI. BMI is the most widely used method to show the increase in fat amount in the whole body. Nevertheless, BMI does not reliably reflect the body fat composition as only body weight and length are taken into consideration [121]. Fat distribution and sexual dimorphism further explain the likely existing gender difference [122, 123]. Mathematician Nick Trefethen believes that the body mass index formula traditionally used to work out if someone is overweight is flawed, and he has come up with his own formula. And he found short people are actually more overweight than they think they are, while tall people are not as overweight as they are being told (Daily Mail. PUBLISHED: 00:08 GMT, January 21, 2013). He claimed a new method for calculating BMI, but again only body height and weight are considered as reference factors. Probably along with waist and neck girth, simple anthropometric measures could amend the weakness of BMI as a single indicator of body size.

 We hereby recommend future research suggestions regarding determining the safe needling depth. Firstly, an international congress should be convened to reach unanimous agreement on the definition of safe needling depth of each acupuncture point and acupuncture point localization method for the future study design. Secondly, factors like gender, age, BMI (or other index to differentiate body sizes), right/left side of the limb, insertion angle, and de-qi should be controlled as much as possible. Subjects (maybe crossing races) should be as many as possible, and the study design should better be randomized control studies. We should try our best to cover every acupuncture point in the whole body. Thirdly, in vivo research is better than retrospective images or specimen dissections. MRI seems to be a better tool to obtain more detailed information of anatomical structures surrounding the acupuncture needle, especially the soft tissue. Fourthly, we also suggest that multicenter should collaboration be carried out to collect statistically valuable information that can be used to increase the safety of acupuncture. Difference among different measuring methods can be understood better.

When it comes to the discussion of clinical effect of acupuncture, de-qi is frequently mentioned. De-qi means a sensation that is often elicited to enhance the effect of acupuncture treatment [124, 125]. In the ancient acupuncture literature (Huangdi) Neijing chapter Suwen indicated that de-qi may have the root in subcutaneous tissue, connective tissues, and muscles layers according to variable conditions including severity of diseases. Some practitioners of acupuncture refer to de-qi as “needle grasp,” a biomechanical phenomenon characterized by an increase in the force necessary to pull the needle out of the tissue. It has been proposed that the sensation of needle grasp is due to the contraction of skeletal muscle [126] or winding of connective tissue around the needle during needle rotation [127]. By using electric impedance, Lin showed that muscle layers were the major site of de-qi in the 22 acupoints in the back [128]. The role of the nervous system in de-qi has also been well described, and some researches in this review shared their opinions as well [24, 129]. The relevant nerves can be found in certain region surrounding the acupuncture point, and it is likely that nerves are provoked during needle manipulation. As discrepancy in definition, mechanism, and location of de-qi still exists, the depth to elicit de-qi in each point and the correlation with clinical efficacy warrants, further researches.

Only limited researches addressed the needling depth with therapeutic efficacy with good designs, and were not all acupuncture points were investigated thoroughly. Lin conducted a meta-analysis of the published evidence concerning acupoint needling depths, with the aim of providing a uniform guidance. Their group's CT scanning results indicate safe needling depths for acupoints in the back and chest for different-sized people, that is, normal, over- and underweight adults, and for sex differences (Tables 7, 8, 9, and 10) [130].

 Some clinicians have shared their clinical experiences about the optimal needling depth of to treat some diseases [131]. As there are still a lot of confusions in needling depth of acupuncture points from the ancient times to the present, which has negatively influenced the standardization and international exchanges of acupuncture science, it remains to be settled as soon as possibly [132]. After convinced that acupuncture is inherently safe, how to avoid the possible associated adverse events and complications is a top priority. We should put more efforts on how to reduce the risk by starting exploring the safe needling depth as well as the depth of clinical efficacy efficiently and correctly.

## 5. Conclusion

From the current review we found that there is great inconsistency in terms of safe needling depths measured from different subject groups and via different measuring tools. The depths measured in each research were somehow influenced by the different measuring methods as they all have distinguished advantages/disadvantages as compared with one another. The results of related researches fail to provide the solid support to decide the best measuring tool among conventional cadaver specimens, CT, MRI or ultrasound either. The characteristics of subjects such as ethnicity, gender, age, body size, underlying diseases, and the needling details (such as needling angle, which anatomical region of the body, which side of the body, and if with de-qi) all contributed partially to the measured depths. The definition of safe depth and standard localization method should be established by standardization through international conference. There is also lack of well-designed researches to compare the therapeutic effects thus making the proper needling depth of clinical efficacy in each acupuncture point remain obscure. A well-designed clinical trial (to control variable strictly, recruit more subjects, etc.) to decide the actual and advisable needling depth for each acupuncture point, to avoid adverse effects or complications and promote optimal clinical efficacy, is a top priority. In vivo MRI imaging may serve as a good study method. 

Different levels of treatment assessment of acupuncture then can be suggested by European Information Centre on complementary and Alternative Medicine (EICCAM II), that is, efficacy (is it more effective than placebo or standard?), effectiveness (is it helpful in usual care?) and efficiency (how is the cost benefit relation?). 

## Figures and Tables

**Table 1:** Summary of researches using MRI images for measurement of the depths of acupuncture points.

Authors and year	Subjects and sample size	Investigated acupuncture points and their body regions/meridians	Parameters used in comparison and related factors	Results, suggestions, and conclusions
Li 2011 [8]	10 male, 10 female	BL18	Gender, angle, side, BL, BW, BMI, and thumb cun	(1) Male subjects had greater safe depth (mean 25.1 mm versus 22.52 mm) (2) Right side points had greater depths (3) The perpendicular depths of right side points correlated with BW, thumb cun; depths of left side points correlated with BL, BW, BMI, and thumb cun

Fu 2011 [9]	10 male, 10 female	BL20	Gender, angle, side, BL, BW, and BMI	(1) No side differences (2) With gender differences except when needling angle is 45 degrees (3) The safe depth did not correlate with BL, BW, and BMI

Chuan 2011 [10]	10 male, 10 female	BL19	Gender, angle, BL, BW, and BMI	(1) The male subject had greater safe depth of perpendicular needling (2) If needled with the angles of 30 and 45 degrees, no gender difference noted (3) The depth may vary profoundly under different angles, for example, 2.02 to 4.0 cm

Yen 2011 [11]	10 male, 10 female	BL17	Gender, angle, side, BL, BW, and BMI	(1) No obvious side difference (2) The various needling angles and safe depth did not correlate with BMI (3) Mean depth of perpendicular depth in male is 4.11 cm and 3.16 cm in females

Han 2010 [12] and Wen et al. 2011 [13]	10 male, 10 female	SP21	Gender, angle, BL, BW, BMI, body cun	(1) The safe needling angle should be about 15 degrees towards the skin surface (2) Difference in safe depth existed in various needling angles (3) The safe depth did not correlate with BL, BW, BMI, thumb cun, and body cun

Yu 2010 [14]	10 male, 10 female	CV14, ST19	Gender, angle, BL, BW, BMI, and body cun	(1) No gender differences in both points (2) the safe depth of CV14 did not correlate with BL, BW, BMI, or body cun (3) the safe depth of ST19 correlated with BW, BMI

Ho 2010 [15]	10 male, 10 female	ST18, GB24	Gender, angle, side, and BMI	(1) No side difference for ST18 (2) Female subjects had greater depths generally for ST18 (3) Safe depths for ST18 and GB24 correlated significantly with BMI

Wu 2010 [16]	10 male, 10 female	LR14	Gender, angle, side, and BMI	(1) Perpendicular needling depth was greater in female subjects (2) No gender difference (3) Dangerous depth of LR14 correlated with BMI significantly

Dong 2010 [17] and Cheng and Dong 2012 [18]	10 male, 10 female	CV15, CV13	Gender, angle, BL, BW, BMI, and body cun	(1) The safe depth for CV15 ranged from 16.99 to 53.47 mm with no gender difference (2) The safe depth for CV13 ranged from 17.25 to 62.74 mm with no gender difference (3) The safe depth of both points did not correlate with BL, BW, and body cun

Wang 2009 [19]	11 male, 9 female	BL10	Gender, side, angle, BL, BW, neck girth, and thumb cun	(1) No side and angle differences noted in both groups (2) Male subjects had greater depth (3) The safe depth did not correlate with any of the parameters

Chang 2009 [20]	11 male, 9 female	GB20	Gender, angle, BL, BW, BMI, neck girth, and head girth	(1) The needling direction towards the nose tip would be the safest way (2) Mean safe depth in males ranged from 35.25 to 42.75 mm while 29.92 to 36.19 in females (3) Safe depth correlated with neck, head girth, BW, and BMI, but needling angle did not

Bai 2009 [21]	11 male, 9 female	GV16	Gender, angle, BL, BW, BMI, neck girth, and thumb cun	(1) Male subjects had greater depth with various needling angles (2) Needling depth correlated positively with BW, BMI, and neck girth (3) Safe depth ranged 27.08–45.55 mm

Wu 2009 [22]	11 male, 9 female	BL1, ST1	Gender	(1) The safe depth was defined as the 75% of dangerous depth (2) Perpendicular needling depth for BL1 was 4.14 mm (19.87 mm if along the axis of eyeball) and 17.85 for ST1 (3) With gender differences

Lu 2009 [23]	11 male, 9 female	GV15	Gender, angle, side, BL, BW, BMI, neck girth, and thumb cun	(1) Perpendicular needling depth was greater in males (2) Oblique needling depth was greater in females at an angle of around 15 degrees (3) Depth in male subjects correlated with BL, BW, and neck girth while depth in female group correlated with none of those parameters

**Table 2:** Summary of researches using in vivo evaluation methods in real subjects.

Authors and year	Subjects and sample size	Investigated acupuncture points and their body regions/meridians	Parameters used in comparison and related factors	Results, suggestions, and conclusions
Groenemeyer et al. 2009 [24]	58 patients with low back pain	BL25, BL26	BMI	(1) An association between de-qi and needle location existed (2) The distance between BL25 and BL6 to the vertebral line was 3.49 ± 0.58 and 3.32 ± 0.53 cm, respectively (3) There was a significant correlation between the interscapular distance and the thickness of the soft tissue layer with the BMI at both acupuncture points

Streitberger et al. 2007 [25]	50 patients receiving acupuncture including PC6 bilaterally (97 wrists)	PC6	Nerve penetrated or contacted	(1) Association between nerve contact and de-qi was discussed. De-qi was elicited in 85 cases. No association between the number of nerve contacts and de-qi was found (2) The mean distance from the needle tip to the nerve was 1.8 mm (standard deviation 2.2; range 0–11.3). Nerve contacts were recorded in 52 cases, in 14 of which the nerve was penetrated by the needle

Dong et al. 2004 [26]	32 adults and 10 cadavers	7 points from bladder meridian (2nd side line)	Rohrer index: <1.2, 1.2–1.5, and >1.5, side	(1) No side difference (2) Depths from in vivo CT images were greater than ones from cadavers (3) Safe depth should be less than 70% of dangerous depth

Li et al. 2004 [27]	32 adults and 10 cadavers	7 points from bladder meridian (1st side line)	Rohrer index: <1.2, 1.2–1.5, and >1.5, side, and needling angles	(1) No side difference (2) Depths from in vivo CT images were greater than ones from cadavers (3) Safe depth should be less than 70% of dangerous depth (4) Safe needling angle should be 10 degrees more than dangerous angle

He et al. 2004 [28]	40 patients of HIVD of C spine	GV14	BL, BW, and AW	(1) Depth ranges 36–75 mm with a mean of 54.6 mm. The safe depth should be within 36 mm (2) Measured depth was greater than documents from ancient writings

Lin 1997 [29]	80 cadavers (including 30 newborns) and 240 adults for safety depth; 300 real subjects for de-qi depth	all back bladder meridian points and chest points	Gender, Tong Shen Cun, BL, BW (normal, over- and underweight) DQ, and AW	(1) Depths were deeper as compared to ancient writings. The depths highly correlated with body thickness and Tong Shen Cun (2) De-qi depth was related to therapeutic effect (3) De-qi depths of chest points were greater in females but not in back points

Lin and Wang 1994 [30]	300 adults	Total of 75 acupoints in head, neck, trunk and lower limb	Gender, BW, and DQ	(1) Discussed de-qi depth but not safe depth (2) Depth of de-qi was greater in males and people with greater body weight (3) Depths in neck region were more superficial in trunk and limbs

Lin 1991 [31]	107 adults	Acupoints in the chest and back of subjects receiving acupuncture therapy	Gender, BW (normal, over- and underweight), BL, and DQ	(1) Overweight group had the greatest de-qi depth (2) Points of female chest had greater depth than male (3) No correlations between the de-qi depth and electric resistance of each point

**Table 3:** Summary of researches using CT scan images for measurement of the depths of acupuncture points.

Authors and year	Subjects and sample size	Investigated acupuncture points and their body regions/meridians	Parameters used in comparison and related factors	Results, suggestions, and conclusions
Chen et al. 2009 [32]	204 pediatric patients aged 7–15	12 abdominal acupuncture points CV-3, CV-4, CV-6, CV-10, CV-12, CV-14, KI-12, ST-24, ST-25, SP-15 LV-13, and LV-4	Gender, age, BW, and waist girth	(1) Using the therapeutic depth over safety depth ratio (T/S ratio) as the indicator of therapeutic depth (2) No significant difference in the T/S ratio between genders (3) The T/S ratio of these 12 acupuncture points ranged from 0.67 to 0.88 and increased significantly with body weight, age, and waist girth (4) The therapeutic depth of abdominal acupoints was closer to the safe depth in overweight and older children aged 7 to 15 (5) No significant difference between genders

Groenemeyer et al. 2009 [24]	58 patients with low back pain	BL25, BL26	BMI	(1) An association between de-qi and needle location existed (2) The distance between BL25 and BL6 to the vertebral line was 3.49 ± 0.58 and 3.32 ± 0.53 cm, respectively (3) There was a significant correlation between the interscapular distance and the thickness of the soft tissue layer with the BMI at both acupuncture points

Yang et al. 2008 [33]	41 adults	GV16	Rohrer index	(1) The safe needling depth should be less than 75% of the dangerous depth (2) The safe depths of GV16 were different for persons of different somatotypes ranging 27.73–33.39 mm

Chen et al. 2008 [34]	219 pediatric patients aged 7–15	12 acupoints along the conception vessel (CV): CV-2 to CV-7 and CV-9 to CV-14	Gender, age, BW, and waist girth	(1) The safe depth of 12 acupoints significantly increased with age, body weight, and waist girth in pediatric patients aged 7–15 (2) There were large variations of the 12 points among different age and body weight groups (3) The safe depths were 1.3–2.1 times deeper in the 12–15-year-old group than in the 7–9-year-old group and 1.7–3 times deeper in overweight children than in underweight children

Chern et al. 2006 [35]	32 adults	BL13	Rohrer index (<1.2, 1.2–1.5, and >1.5), side	(1) Right side points seemed to be deeper, especially in people with Rohrer index <1 (2) Safety depth should be within 75% of the measured distance in each group; that is, 34, 25, and 23 mm

Li et al. 2005 [36]	32 adults	GV14, SI15, GV5, and GV4	Rohrer index: <1.2, 1.2–1.5, and >1.5	The safe depths (75% of dangerous depths) were different for different somatotypes; for example, the needling depth for GV14 was 32.86 ± 3.96 mm for the thin person group and 47.93 ± 5.30 mm for the fat person group

Dong et al. 2004 [26]	32 adults and 10 cadavers	7 points from bladder meridian (2nd side line)	Rohrer index: <1.2, 1.2–1.5, and >1.5, side	(1) No side difference (2) Depths from in vivo CT images were greater than ones from cadavers (3) Safe depth should be less than 70% of dangerous depth

Li et al. 2004 [27]	32 adults and 10 cadavers	7 points from bladder meridian (1st side line)	Rohrer index: <1.2, 1.2–1.5, and >1.5, side, and needling angles	(1) No side difference (2) Depths from in vivo CT images were greater than ones from cadavers (3) Safe depth should be less than 70% of dangerous depth (4) Safe needling angle should be 10 degrees more than dangerous angle

Lin 1997 [29]	80 cadavers (including 30 newborns) and 240 adults for safety depth; 300 real subjects for de-qi depth	All back bladder meridian points and chest points	Gender, Tong Shen Cun, BL, BW (normal, over- and underweight) DQ, and AW	(1) Depths were deeper as compared to ancient writings. The depths highly correlated with body thickness and Tong Shen Cun (2) De-qi depth was related to therapeutic effect (3) De-qi depths of chest points were greater in females but not in back points

Sheu and Lin 1992 [37]	120 adults	28 points in the chest from conception vessel, kidney meridian, stomach meridian, pericardium meridian, lung meridian, spleen meridian, and gallbladder meridian	gender, BW (normal, over- and underweight), and BL	(1) Significant differences in chest points within the same sex existed (2) For different body sizes, statistically significant differences for each point appeared

Lin et al. 1991 [38]	240 adults (120 in each group)	22 points in the back; 28 points in the chest	Gender, BW (normal, over- and underweight), and BL	(1) No gender differences on back loci (2) Significant differences in each point with different body sizes (3) Female chest points had greater depths (4) The results should be more accurate than cadaver study

**Table 4:** Summary of researches using ultrasound images for measurement of the depths of acupuncture points.

Authors and year	Subjects and sample size	Investigated acupuncture points and their body regions/meridians	Parameters used in comparison and related factors	Results, suggestions, and conclusions
Lian 1995 [79]	89 adults	From BL11 to BL21 (11 points)	Gender, age, BL, BW, disease type, side, and AW	(1) Depths ranged 12–40 mm (2) Depth was not affected by age or body sizes (3) Points of male subjects and right side of the body had greater depths (4) Disease type did not affect depth (5) Depths measured were shorter compared to ancient writings

Streitberger et al. 2007 [25]	50 patients receiving acupuncture including PC6 bilaterally (97 wrists)	PC6	Nerve penetrated or contacted DQ	(1) Association between nerve contact and de-qi was discussed. De-qi was elicited in 85 cases. No association between the number of nerve contacts and de-qi was found (2) The mean distance from the needle tip to the nerve was 1.8 mm (standard deviation 2.2; range 0–11.3). Nerve contacts were recorded in 52 cases, in 14 of which the nerve was penetrated by the needle

**Table 5:** Summary of researches using dissected specimens of cadavers for measurement of the depths of acupuncture points.

Authors and year	Subjects and sample size	Investigated acupuncture points and their body regions/meridians	Parameters used in comparison and related factors	Results, suggestions, and conclusions
Oh et al. 2012 [80]	4 adult cadavers	PC6	—	(1) The acupuncture needle should not be inserted deeply at PC6 in order to minimise the risk of trauma (2) It seemed likely that various kinds of noninvasive stimulation at PC6 may be similarly effective as needling (3) Careful insertion of the needle with respect to patients' sensations and better anatomical knowledge of the forearm can help to prevent unexpected needle penetration of the median nerve or persistent median artery

Wang et al. 2009 [81]	15 adult cadavers	ST7, SI18	Side	(1) No side difference (2) Mean inserting depth of ST7 and SI18 to sphenopalatine ganglion were 49.9 and 46.6 mm

Xie et al. 2007 [82]	46 adult cadavers	SI14 and GV14	Gender	(1) No gender difference (2) The mean dangerous depth for perpendicular insertion was 60.60 mm for SI14 and 55.93 mm for GV14. (3) Suggested depth for perpendicular needling of SI14 and GV14 was within 42 mm in adult

Chen et al. 2007 [83]	46 cadavers	CV22, ST11	—	(1) The needle not only easily injuried the upper pleural cavity but also damaged the big blood vessel, the vagus nerve in the mediastinum and the cervical root (2) The safety depth of ST11 ranged 23.7–52.8 mm

Xie et al. 2006 [84]	46 cadavers	ST12	Gender, side	(1) No gender or side difference (2) The mean dangerous depth of male was 34.97 mm and female 31.41 mm (3) The depth for perpendicular needling of SI12 is within 22.50 mm

Xie et al. 2006 [85]	46 cadavers	BL12, BL13	Gender, angles of needle insertion (15, 20, 25, 30, and 40 degrees)	(1) The mean dangerous depth for perpendicular insertion was 49.51 mm of BL12 and 44.88 mm of BL13 (2) It was safe for oblique insertion toward the medial of chest in an angle exceeding 20 degrees (3) No gender difference

Xu et al. 2006 [86]	48 adult cadavers	BL1	—	(1) The mean depth between the skin and the anterior ethmoidal artery was 18.25 ± 4.45 mm, with an angle of 12.5 ± 5.5 degrees (2) The depth from the skin to the optic nerve tunnel frontal point was 43.37 ± 7.84 mm (3) Needling depth should not exceed 30.36 mm (70% of measured depth) to avoid injury of the optic nerve

Lou et al. 2006 [87]	80 limbs (40 cadavers)	ST36	Angle	(1) Average depth was 2.22 cm (2) Maximal depth was 4.42 cm if needled obliquely (3) Safe depth was generally less than 5 cm

Chen et al. 2006 [88]	46 cadavers	CV22, ST11, ST12, GB21, EX-B1, and BL11	—	(1) Risk of pleural injury may existed when inserting needle perpendicularly in these points (2) Divergence existed in observed needling depths such as 22.5–61.3 mm for ST12

Dong et al. 2004 [26]	32 adults and 10 cadavers	7 points from bladder meridian (2nd side line)	Rohrer index: <1.2, 1.2–1.5, and >1.5, side	(1) No side difference (2) Depths from in vivo CT images were greater than ones from cadavers (3) Safe depth should be less than 70% of dangerous depth

Li et al. 2004 [27]	32 adults and 10 cadavers	7 points from bladder meridian (1st side line)	Rohrer index: <1.2, 1.2–1.5, and >1.5, side, and needling angles	(1) No side difference (2) Depths from in vivo CT images were greater than ones from cadavers (3) Safe depth should be less than 70% of dangerous depth (4) Safe needling angle should be 10 degrees more than dangerous angle

Yan et al. 2004 [89]	51 cadavers	74 points from neck, chest, back, and abdomen	Angles of insertion	(1) Safe depth should be less than 70% of dangerous depth (2) Needling angles were suggested such as 65 degrees rather than perpendicular insertion for points in the bladder meridian

Zhang et al. 2001 [90]	51 cadavers	17 acupoints of abdomen	Gender, side	(1) The dangerous depth of most abdominal points were similar and within 11–17 mm (2) KI11 had the greatest depth up to 25 mm (to the urinary bladder) but with divergence in standard error

Zhang et al. 2001 [91]	57 cadavers	ST12	—	(1) The mean dangerous depths for perpendicular insertion downward was 38.34 mm (2) Safety depth was within 26.83 mm (70% of the dangerous depth)

Piao and Zhong2001 [92]	6 cadavers	B2 (lumbar levels)	different lumbar vertebrae: L1–L5	The depth ranged 4.2–5.75 cm in different lumbar levels with greater depths in lower lumbar levels

Chen et al. 1998 [93]	20 adult cadavers	BL40	Side	(1) Safe depth (from skin to tibial nerve): 15 mm for left side and 16 mm for right side, less than the depth from current used textbook (2) Depth from skin to deep vein: 35 mm

Ge 1998 [94]	16 cadavers	GV15, GV16	Thumb Tong Shen Cun	Safe depth of GV15 (42.46–55.86 mm) and GV16 (43.46–57.42 mm) correlated with thumb Tong Shen Cun

Zhang et al. 1998 [95]	51 cadavers	Total of 28 acupoints in back and lumbar region	Gender, side	(1) No side difference in dangerous depth except BL17, BL18 for male and BL17 for female (2) Points of bladder meridian closer to the spine had greater depths. Divergence existed between points (3) There was no gender/side difference

Zhang et al. 1998 [96]	51 cadavers	23 chest points	Gender, side	(1) The average dangerous depths of 23 chest acupoints were obtained. KI27 had the greatest dangerous depth up to 26 mm. Others ranged from 11.87 to 17.64 mm (2) Divergence existed between points (3) There was no gender/side difference (4) Safe depth should be less than 70% of dangerous depth

Lin 1997 [29]	80 cadavers (including 30 newborns) and 240 adults for safety depth; 300 real subjects for de-qi depth	All back bladder meridian points and chest points	Gender, Tong Shen Cun, BL, BW (normal, over- and underweight) DQ, and AW	(1) Depths were deeper as compared to ancient writings. The depths highly correlated with body thickness and Tong Shen Cun (2) De-qi depth was related to therapeutic effect (3) De-qi depths of chest points were greater in females but not in back points

Yan et al. 1996 [97]	51 cadavers	GV16, GV15, GB20, and BL1	Gender, side	(1) The safe depths (80% of the measured depth) were GV16: 40.08 mm, GV15: 38.10 mm, GB20: 39.77 mm, and BL1: 34.25 mm (2) No gender or side difference

**Table 6:** Summary of researches involving the correlation between therapeutic effect and needling depth.

Authors and year	Subjects and sample size	Investigated acupuncture points and their body regions/meridians	Parameters used in comparison and related factors	Results, suggestions, and conclusions
He et al. 2012 [98]	63 subjects with trigeminal neuralgia (32 in deep and 31 in shallow puncturing group)	ST7, LI4, LV3, BL2, ST2, and Jiacheng Jiang	Pain index (VAS), traditional Chinese medicine symptoms index, and clinical therapeutic effect	(1) The total effective rate was 93.8% in deep puncturing group, superior to that of 87.1% in shallow puncturing group (2) No adverse reaction was observed in both groups (3) Deep puncturing at ST7 to the depth of sphenopalatine ganglion was more effective than routine puncturing

Park et al. 2011 [99]	5 participants	LI13, LU4 and 2 control points	Needling depth, needle rotation, and oscillation	(1) Pilot study using ultrasound tried to explore the correlation between de-qi sensation and needling depth/needle manipulation (2) Shallower insertion induced more sharp sensations (3) Deeper insertion induced more dull sensations (4) Needle rotation significantly increased the dull sensations

Skjeie et al. 2011 [100]	7 randomized patients (3 in placebo group and 4 in acupuncture treatment group)	ST36	Bilateral insertion at ST36 at the depth of 12 mm, reduction of crying time from baseline	(1) A pilot, open, randomized, and single-blinded controlled trial to assess the feasibility of acupuncture treatment for infantile colic (2) No adverse events were reported (3) Acupuncture group had more reduction of crying time from baseline

Lu and Tang 2011 [101]	21 cases of irritable bowel syndrome of diarrhea	IR13, CV12, ST25, CV4, LR14, LI11, LI4, SP9, ST36, and LR3	The scale for the severity degree of symptom (IBS-SSS)	(1) Various needling depths ranged 2–12 mm as documented in Lingshu (Miraculous Pivot) (2) After treatment, there was significant change in IBS-SSS, and the effective rate may reach 90.5% (3) The longer the session of treatment was, the better the efficacy was obtained (4) Confirmed the needling depth recorded in Lingshu

Itoh et al. 2011 [102]	22 healthy volunteers	Tender point in the extensor digital muscle, in the skin, and in the nonsegmental limb (anterior tibial muscle)	Pressure pain threshold, electrical pain threshold, and needling depth (3 mm to 10 mm)	(1) Randomized controlled trial (2) Immediate pain relief in muscle group (depth of 10 mm insertion into extensor digital muscle) which was better than skin group (depth of 3 mm) (3) Acupuncture stimulation of muscle increases the PPT and EPT of fascia. The depth of needle penetration was important for the relief of muscle pain

Chen et al. 2009 [32]	204 pediatric patients aged 7–15	12 abdominal acupuncture points CV-3, CV-4, CV-6, CV-10, CV-12, CV-14, KI-12, ST-24, ST-25, SP-15 LV-13, and LV-4	Gender, age, BW, and waist girth	(1) Using the therapeutic depth over safety depth ratio (T/S ratio) as the indicator of therapeutic depth (2) No significant difference in the T/S ratio between genders (3) The T/S ratio of these 12 acupuncture points ranged from 0.67 to 0.88 and increased significantly with body weight, age, and waist girth (4) The therapeutic depth of abdominal acupoints was closer to the safe depth in overweight and older children aged 7 to 15 (5) No significant difference between genders

Groenemeyer et al. 2009 [24]	58 patients with low back pain	BL25, BL26	BMI	(1) An association between de-qi and needle location existed (2) The distance between BL25 and BL6 to the vertebral line is 3.49 ± 0.58 and 3.32 ± 0.53 cm, respectively (3) There was a significant correlation between the interscapular distance and the thickness of the soft tissue layer with the BMI at both acupuncture points

Streitberger et al. 2007 [25]	50 patients receiving acupuncture including PC6 bilaterally (97 wrists)	PC6	Nerve penetrated or contacted DQ	(1) Association between nerve contact and de-qi was discussed. De-qi was elicited in 85 cases. No association between the number of nerve contacts and de-qi was found (2) The mean distance from the needle tip to the nerve was 1.8 mm (standard deviation 2.2; range 0–11.3). Nerve contacts were recorded in 52 cases, in 14 of which the nerve was penetrated by the needle

Lin 1997 [29]	80 cadavers (including 30 newborns) and 240 adults for safety depth; 300 real subjects for de-qi depth	All back bladder meridian points and chest points	Gender, Tong Shen Cun, BL, BW (normal, over and under-weight) DQ, and AW	(1) Depths were deeper as compared to ancient writings. The depths highly correlated with body thickness and Tong Shen Cun (2) De-qi depth was related to therapeutic effect (3) De-qi depths of chest points were greater in females but not in back points

Lin and Wang 1994 [30]	300 adults	Total of 75 acupoints in head, neck, trunk, and lower limb	Gender, BW, and DQ	(1) Discussed de-qi depth but not safe depth (2) Depth of de-qi was greater in males and people with greater body weight (3) Depths in neck region were more superficial in trunk and limbs

Lin 1991 [31]	107 adults	Acupoints in the chest and back of subjects receiving acupuncture therapy	Gender, BW (normal, over- and underweight), BL, and DQ	(1) Overweight group had the greatest de-qi depth (2) Points of female chest had greater depth than male (3) No correlations between the de-qi depth and electric resistance of each point

**Table 7:** Mean values for needling depths of acupoints in the chest in different-sized male subjects.

Acupoints	Overweight adults	Normal adults	Underweight adults	*F*
	Mean ± 95% C.I.	Mean ± 95% C.I.	Mean ± 95% C.I.	
Tiantu (CV22)	3.94 ± 0.19	2.87 ± 0.26	2.34 ± 0.65	19.61*
Xuanji (CV21)	0.94 ± 0.11	0.64 ± 0.10	2.30 ± 0.10	38.22*
Huagai (CV20)	0.89 ± 0.11	0.057 ± 0.10	0.25 ± 0.10	39.46*
Zigong (CV19)	0.89 ± 0.11	0.60 ± 0.10	0.29 ± 0.10	35.79*
Yutang (CV18)	0.88 ± 0.14	0.53 ± 0.10	0.28 ± 0.09	31.32*
Danzhong (CV17)	0.86 ± 0.11	0.52 ± 0.11	0.25 ± 0.09	36.96*
Zhongting (CV16)	0.95 ± 0.14	0.56 ± 0.13	0.34 ± 0.11	25.57*
Shufu (KI27)	4.19 ± 0.46	3.29 ± 0.48	2.32 ± 0.49	16.20*
Yuzhong (KI26)	2.98 ± 0.27	2.20 ± 0.25	1.50 ± 0.27	34.18*
Shencang (KI25)	2.59 ± 0.21	2.00 ± 0.21	1.26 ± 0.22	42.93*
Lingxu (KI24)	2.56 ± 0.17	2.13 ± 0.23	1.49 ± 0.19	32.09*
Shenfeng (KI23)	2.47 ± 0.17	1.96 ± 0.17	1.44 ± 0.17	40.78*
Bulang (KI22)	2.33 ± 0.15	1.95 ± 0.17	1.46 ± 0.15	33.64*
Qihu (ST13)	5.24 ± 0.48	4.15 ± 0.55	2.88 ± 0.47	23.71*
Kufang (ST14)	3.82 ± 0.36	3.10 ± 0.31	2.02 ± 0.29	32.21*
Wuyi (ST15)	3.11 ± 0.27	2.64 ± 0.52	1.38 ± 0.17	26.51*
Yingchuang (ST16)	2.78 ± 0.21	2.35 ± 0.53	1.33 ± 0.22	19.52*
Ruzhong (ST17)	2.59 ± 0.19	2.07 ± 0.38	1.23 ± 0.20	27.28*
Rugen (ST18)	2.27 ± 0.16	1.78 ± 0.22	1.19 ± 0.20	33.89*
Tianchi (PC1)	2.64 ± 0.21	2.25 ± 0.72	1.18 ± 0.21	11.97*
Yunmen (LU1)	6.73 ± 0.55	5.14 ± 0.57	3.26 ± 0.79	32.78*
Zhongfu (LU2)	5.05 ± 0.62	3.69 ± 0.47	2.20 ± 0.70	24.06*
Zhourong (SP20)	3.71 ± 0.50	2.70 ± 0.35	1.68 ± 0.51	21.06*
Xiongxiang (SP19)	3.15 ± 0.37	2.26 ± 0.23	1.53 ± 0.43	23.38*
Tianxi (SP18)	2.88 ± 0.29	2.05 ± 0.25	1.32 ± 0.19	40.68*
Shidou (SP17)	2.61 ± 0.21	1.91 ± 0.22	1.28 ± 0.18	45.01*
Zhejin (GB23)	3.48 ± 0.39	2.43 ± 0.26	1.73 ± 0.37	28.00*
Yuanye (GB22)	4.52 ± 0.41	3.07 ± 0.34	2.20 ± 0.33	43.58*

*X*: mean depth; units are provided in centimeters.

*X* ± 1.96 SD: 95% confidence interval.

*P < 0.01; *F* is the statistic for one-way ANOVA.

The definitions for “Overweight adults,” “Normal adults,” and “Underweight adults” are following the guidance of the Department of Health, Taiwan: “The suggested ideal body weight of Taiwanese people.” As such, readers from outside Taiwan should bear in mind that ideal body weights differ between countries. The specified needling depths in the table are a suggested guide only.

**Table 8:** Mean values for needling depths of acupoints in the chest in different-sized female subjects.

Acupoints	Overweight adults	Normal adults	Underweight adults	*F*
	Mean ± 95% C.I.	Mean ± 95% C.I.	Mean ± 95% C.I.	
Tiantu (CV22)	4.46 ± 0.42	3.69 ± 0.46	3.01 ± 0.80	7.39*
Xuanji (CV21)	1.37 ± 0.27	1.03 ± 0.25	0.41 ± 0.33	9.81*
Huagai (CV20)	1.18 ± 0.29	0.88 ± 0.23	0.49 ± 0.38	5.24*
Zigong (CV19)	1.32 ± 0.24	0.97 ± 0.19	0.50 ± 0.40	9.73*
Yutang (CV18)	1.42 ± 0.25	1.00 ± 0.20	0.56 ± 0.46	9.89*
Danzhong (CV17)	1.55 ± 0.23	1.07 ± 0.22	0.71 ± 0.57	8.63*
Zhongting (CV16)	1.75 ± 0.003	1.41 ± 0.32	0.90 ± 0.76	4.17*
Shufu (KI27)	4.31 ± 0.72	3.36 ± 0.47	2.23 ± 0.46	9.90*
Yuzhong (KI26)	2.93 ± 0.44	2.46 ± 0.33	1.53 ± 0.55	9.31*
Shencang (KI25)	2.69 ± 0.39	2.29 ± 0.29	1.53 ± 0.46	8.35*
Lingxu (KI24)	2.70 ± 0.35	2.40 ± 0.26	1.69 ± 0.57	7.19*
Shenfeng (KI23)	2.89 ± 0.32	2.46 ± 0.24	1.79 ± 0.60	9.52*
Bulang (KI22)	2.95 ± 0.30	2.49 ± 0.23	1.79 ± 0.45	11.15*
Qihu (ST13)	4.73 ± 0.77	3.75 ± 0.57	2.46 ± 1.10	8.14*
Kufang (ST14)	3.51 ± 0.51	3.04 ± 0.45	2.06 ± 1.07	5.52*
Wuyi (ST15)	3.05 ± 0.52	2.72 ± 0.38	1.76 ± 0.82	5.61*
Yingchuang (ST16)	2.94 ± 0.47	2.66 ± 0.37	1.90 ± 0.78	4.08*
Ruzhong (ST17)	2.91 ± 0.42	2.58 ± 0.33	1.93 ± 0.62	4.73*
Rugen (ST18)	2.9200 ± 0.4038	2.444 ± 0.3052	1.6857 ± 0.5636	9.4451*
Tianchi (PC1)	3.34 ± 0.69	2.86 ± 0.37	1.89 ± 0.73	5.37*
Yunmen (LU1)	5.84 ± 0.79	4.42 ± 0.76	3.51 ± 1.59	6.52*
Zhongfu (LU2)	4.43 ± 0.55	3.74 ± 0.47	2.81 ± 1.45	5.39*
Zhourong (SP20)	3.82 ± 0.59	3.49 ± 0.46	2.37 ± 1.15	4.72*
Xiongxiang (SP19)	3.70 ± 0.53	3.31 ± 0.42	2.23 ± 0.82	6.40*
Tianxi (SP18)	3.41 ± 0.53	3.06 ± 0.44	2.07 ± 0.79	5.25*
Shidou (SP17)	3.34 ± 0.53	2.80 ± 0.40	1.83 ± 0.72	7.49*
Zhejin (GB23)	3.63 ± 0.57	3.43 ± 0.43	2.24 ± 0.99	5.24*
Yuanye (GB22)	4.06 ± 0.64	3.72 ± 0.54	2.39 ± 1.08	5.48*

*X*: mean depth; units are provided in centimeters.

*X* ± 1.96 SD: 95% confidence interval.

*P < 0.01; *F* is the statistic for one-way ANOVA.

The definitions for “Overweight adults,” “Normal adults,” and “Underweight adults” are following the guidance of the Department of Health, Taiwan: “The suggested ideal body weight of Taiwanese people.” As such, readers from outside Taiwan should bear in mind that ideal body weights differ between countries. The specified needling depths in the table are a suggested guide only.

**Table 9:** Mean values for needling depths of acupoints in the back from different-sized male subjects.

Acupoints	Overweight adults	Normal adults	Underweight adults	*F*
	Mean ± 95% C.I.	Mean ± 95% C.I.	Mean ± 95% C.I.	
Dazhui (GV14)	6.76 ± 0.41	5.39 ± 0.40	4.81 ± 0.54	20.90*
Taodao (GV13)	6.35 ± 0.40	5.24 ± 0.40	4.66 ± 0.47	17.03*
Shenzhu (GV12)	5.39 ± 0.37	4.79 ± 0.35	4.10 ± 0.34	14.13*
Shendao (GV11)	4.86 ± 0.32	4.30 ± 0.30	3.65 ± 0.23	18.95*
Lingtai (GV10)	4.88 ± 0.32	4.27 ± 0.30	3.56 ± 0.21	22.69*
Zhiyang (GV9)	4.86 ± 0.33	4.20 ± 0.27	3.47 ± 0.19	27.32*
Jianzhongshu (SI15)	7.43 ± 0.50	6.47 ± 0.59	5.77 ± 0.70	8.18*
Dazhu (BL11)	6.98 ± 0.54	6.19 ± 0.49	5.36 ± 0.79	7.33*
Fengmen (BL12)	6.21 ± 0.50	5.53 ± 0.47	5.08 ± 0.77	3.95*
Feishu (BL13)	5.70 ± 0.49	5.15 ± 0.48	4.67 ± 0.66	3.73*
Jueyinshu (BL14)	5.37 ± 0.47	4.76 ± 0.41	4.39 ± 0.57	4.42*
Xinshu (BL15)	5.04 ± 0.67	4.54 ± 0.43	4.27 ± 0.50	2.25*
Dushu (BL16)	5.18 ± 0.47	4.52 ± 0.48	4.13 ± 0.45	5.34*
Geshu (BL17)	5.30 ± 0.47	4.55 ± 0.46	4.18 ± 0.47	6.28*
Jianwaishu (SI14)	6.05 ± 0.39	5.39 ± 0.43	5.00 ± 0.63	4.79*
Fufen (BL41)	5.10 ± 0.45	4.37 ± 0.37	4.38 ± 0.59	3.40*
Pohu (BL42)	4.40 ± 0.37	3.75 ± 0.35	3.56 ± 0.50	5.06*
Gaohuang (BL43)	3.98 ± 0.35	3.34 ± 0.35	2.98 ± 0.41	7.99*
Shentang (BL44)	3.75 ± 0.36	2.98 ± 0.30	2.57 ± 0.35	13.39*
Yixi (BL45)	3.70 ± 0.46	2.76 ± 0.28	2.28 ± 0.37	16.03*
Geguan (BL46)	3.66 ± 0.45	2.63 ± 0.28	2.33 ± 0.36	15.08*
Quyuan (SI13)	5.36 ± 0.32	4.76 ± 0.35	4.31 ± 0.42	8.78*

*X*: mean depth; units are provided in centimeters.

*X* ± 1.96 SD: 95% confidence interval.

*P < 0.01; *F* is the statistic for one-way ANOVA.

The definitions for “Overweight adults,” “Normal adults,” and “Underweight adults” are following the guidance of the Department of Health, Taiwan: “The suggested ideal body weight of Taiwanese people.” As such, readers from outside Taiwan should bear in mind that ideal body weights differ between countries. The specified needling depths in the table are a suggested guide only.

**Table 10:** Mean values for needling depths of acupoints in the back in different-sized female subjects.

Acupoints	Overweight adults	Normal adults	Underweight adults	*F*
	Mean ± 95% C.I.	Mean ± 95% C.I.	Mean ± 95% C.I.	
Dazhui (GV14)	6.37 ± 0.71	5.21 ± 0.70	4.35 ± 0.19	5.91*
Taodao (GV13)	6.01 ± 0.45	4.97 ± 0.58	4.13 ± 0.88	8.58*
Shenzhu (GV12)	5.36 ± 0.35	4.40 ± 0.39	3.60 ± 0.64	15.31*
Shendao (GV11)	5.01 ± 0.38	3.97 ± 0.30	3.40 ± 0.64	18.12*
Lingtai (GV10)	4.89 ± 0.35	3.83 ± 0.28	3.28 ± 0.63	21.03*
Zhiyang (GV9)	4.90 ± 0.41	3.86 ± 0.29	3.37 ± 0.67	15.06*
Jianzhongshu (SI15)	6.62 ± 0.48	5.87 ± 0.62	4.15 ± 1.36	10.43*
Dazhu (BL11)	6.42 ± 0.53	5.39 ± 0.62	4.27 ± 0.96	8.77*
Fengmen (BL12)	5.83 ± 0.39	4.78 ± 0.61	4.13 ± 1.08	6.97*
Feishu (BL13)	5.32 ± 0.38	4.43 ± 0.46	4.03 ± 0.97	6.66*
Jueyinshu (BL14)	5.07 ± 0.47	4.25 ± 0.43	3.73 ± 0.87	6.40*
Xinshu (BL15)	4.91 ± 0.51	3.97 ± 0.32	3.63 ± 0.88	8.36*
Dushu (BL16)	4.90 ± 0.49	3.86 ± 0.30	3.63 ± 0.80	10.35*
Geshu (BL17)	4.91 ± 0.47	3.92 ± 0.37	3.58 ± 0.92	8.57*
Jianwaishu (SI14)	5.57 ± 0.41	4.91 ± 0.48	3.85 ± 0.81	8.83*
Fufen (BL41)	4.85 ± 0.31	4.07 ± 0.42	3.07 ± 0.85	12.60*
Pohu (BL42)	4.35 ± 0.28	3.53 ± 0.31	2.60 ± 0.74	19.67*
Gaohuang (BL43)	3.96 ± 0.32	3.15 ± 0.31	2.30 ± 0.61	18.00*
Shentang (BL44)	3.67 ± 0.35	2.75 ± 0.27	2.02 ± 0.48	21.33*
Yixi (BL45)	3.59 ± 0.37	2.56 ± 0.23	1.88 ± 0.54	24.99*
Geguan (BL46)	3.59 ± 0.40	2.53 ± 0.28	1.78 ± 0.49	22.77*
Quyuan (SI13)	5.16 ± 0.37	4.32 ± 0.39	3.08 ± 0.58	18.83*

*X*: mean depth; units are provided in centimeters.

*X* ± 1.96 SD: 95% confidence interval.

*P < 0.01; *F* is the statistic for one-way ANOVA.

The definitions for “Overweight adults,” “Normal adults,” and “Underweight adults” are following the guidance of the Department of Health, Taiwan: “The suggested ideal body weight of Taiwanese people.” As such, readers from outside Taiwan should bear in mind that ideal body weights differ between countries. The specified needling depths in the table are a suggested guide only.
